# SNP and Haplotype-Based Genomic Selection of Quantitative Traits in *Eucalyptus globulus*

**DOI:** 10.3390/plants8090331

**Published:** 2019-09-05

**Authors:** Paulina Ballesta, Carlos Maldonado, Paulino Pérez-Rodríguez, Freddy Mora

**Affiliations:** 1 Institute of Biological Sciences, University of Talca, 2 Norte 685, Talca 3460000, Chile (P.B.) (C.M.); 2 Colegio de Postgraduados, Statistics and Computer Sciences, Montecillos, Edo. de México 56230, Mexico

**Keywords:** genomic prediction, haplotype blocks, predictive ability, Bayesian models

## Abstract

*Eucalyptus globulus* (Labill.) is one of the most important cultivated eucalypts in temperate and subtropical regions and has been successfully subjected to intensive breeding. In this study, Bayesian genomic models that include the effects of haplotype and single nucleotide polymorphisms (SNP) were assessed to predict quantitative traits related to wood quality and tree growth in a 6-year-old breeding population. To this end, the following markers were considered: (a) ~14 K SNP markers (SNP), (b) ~3 K haplotypes (HAP), and (c) haplotypes and SNPs that were not assigned to a haplotype (HAP-SNP). Predictive ability values (PA) were dependent on the genomic prediction models and markers. On average, Bayesian ridge regression (BRR) and Bayes C had the highest PA for the majority of traits. Notably, genomic models that included the haplotype effect (either HAP or HAP-SNP) significantly increased the PA of low-heritability traits. For instance, BRR based on HAP had the highest PA (0.58) for stem straightness. Consistently, the heritability estimates from genomic models were higher than the pedigree-based estimates for these traits. The results provide additional perspectives for the implementation of genomic selection in *Eucalyptus* breeding programs, which could be especially beneficial for improving traits with low heritability.

## 1. Introduction

The number of breeding programs that use the principles of genomic selection (GS) has increased considerably in recent years. Over the last decade, several investigations have illustrated how to incorporate the principles of genomic prediction in the genetic improvement programs of complex traits [1,2,3,4,5,6,7,8,9,10]. In this sense, the use of GS principles has been facilitated by the development of genotyping techniques such as genotyping by sequencing (GBS) and DNA arrays (or chips) of high density in various crops including self-pollinated plants such as soybean; wheat; barley; rice [10,11,12,13]; and outcrossing plants such as fruit trees, forest trees, and corn, among others [14,15,16,17]. Single nucleotide polymorphic markers (SNP) have been a powerful tool in breeding programs for different agricultural crops [18,19,20,21,22,23]. SNP markers have multiple applications in plants, including positional cloning, whole genome association studies, the mapping of quantitative trait loci (QTL), and the determination of genetic relationships between individuals.

The non-random association between two or more loci due to a low probability of recombination between them (linkage disequilibrium: LD) in a given population constitutes haplotypes [24,25], which correspond to sets of genomic regions within a chromosome that tend to be inherited together [26]. In this context, GS can be implemented not only using individual SNPs but also haplotypes, or a combination of both, using haplotypes in conjunction with SNPs not assigned to a haplotype. For example, Cuyabano et al. [27] presented a haplotype approach for genomic prediction using high-density data in dairy cattle as an alternative to individual marker methods, demonstrating that the haplotypes improved prediction accuracy compared to an individual SNP. On the other hand, Calus et al. [28] determined that the inclusion of haplotypes in genomic prediction models was beneficial for low-heritability traits. Matias et al. [29] found that the use of haplotypes in the prediction of complex traits of maize increased the predictive ability by 20%. From these and other studies, it appears that the haplotype approach emerges as a methodological variant that can improve not only the predictive abilities but also the precision in the detection of genomic regions in association studies [30,31,32,33]. Moreover, given that a haplotype is defined as a set of nearby SNPs, which are in strong LD [19], this analytical approach would take the natural dependence that exists between SNPs into account, which becomes more relevant when considering high-density DNA arrays.

Several studies have emphasized the use of haplotypes (from high-density DNA arrays) to estimate the predictive ability of different GS models [27,34,35,36,37,38]. One of the advantages of using haplotypes in GS is the ability to detect and include mutations as genomic information. According to Curtis et al. [39], when mutations have occurred, it is possible that the frequencies of the alleles remain (almost) unchanged. However, when analyzing haplotypes, mutations at different loci tend to cause significant changes in haplotype frequencies. Therefore, a QTL that is not in complete LD with an individual marker can be in complete LD with a particular haplotype [27]. Additionally, the use of haplotypes reduces the degrees of freedom in the models of prediction or genomic association (reduction of dimensionality), which contributes to greater precision in the detection of QTL [40]. It should be noted that there are few studies that have evaluated the combined use of GS and haplotypes in plants [29,41,42]. In particular, these methods have been implemented in predominantly self-pollinated (autogamous) species—for example, soybean and wheat [41,42]—in which extensive LD values can be found in their genomes, which favors the identification of haplotypes, while in outcrossing species (allogamous) such as *Eucalyptus* and most species of forest interest, LD usually decays at short genomic distances [43,44], which allows the identification of smaller haplotype blocks and those conformed by a smaller number of alleles.

The objectives of this study were to (i) determine and characterize haplotype blocks in *Eucalyptus globulus* from a high-quality SNP array (EUChip60K), (ii) investigate the possible benefits of using haplotype information to predict complex traits in trees, and (iii) estimate genomic parameters (e.g., genomic heritability and genetic gains) using Bayesian whole-regression models (Bayesian Ridge Regression, Bayes A, Bayes B, Bayes C and Bayesian Least Absolute Shrinkage and Selection Operator) that include the haplotype/SNP effect on a 6-year-old breeding population of *E. globulus*.

## 2. Results

### 2.1. Haplotype Block Construction

The final dataset used for the haplotype block construction and genomic predictions consisted of 14,422 SNPs. An average of 1356 SNPs per chromosome and an average frequency of one SNP every 4000 bp were found. Chromosome 8 contained 12.5% (1811 SNPs) of the total SNPs, while chromosome 4 presented only 6.2% (893 SNPs). Total values of 1137 haplotype blocks and 3279 haplotypes (Table 1) were identified in all chromosomes of the species under study. In total, 14.5% of the total SNPs (2092 SNPs) were grouped into haplotype blocks. The largest number of haplotype blocks was determined by combinations of SNPs located on chromosome 8 (*n* = 152), while a smaller amount was constructed by SNPs located on chromosome 4 (*n* = 71). Of the total blocks formed by the chromosomes, 300 haplotypes were obtained (on average) per chromosome, while each block had three haplotype variants on average. The number of SNPs within each haplotype block varied from two to 12. The smallest blocks had an extension of 36 bp, while the largest haplotypes had a length of 482 kbp (chromosome 8). About 24% of the total haplotype blocks had a size greater than 10 kbp, while 2% had a size above 100 kbp. Particularly, on chromosomes 4 and 9, no haplotype blocks with an extension higher than 100 kbp were detected. The genome-wide average linkage disequilibrium decay estimated across all chromosomes is shown in Figure S1 (Supplementary Material). On a genome-wide average, LD decayed within ~10–12 Kbp to a level below *r*^2^ = 0.14 (critical *r*^2^ value).

### 2.2. Estimates of Genetic Parameters Based on Pedigree

Estimates of variance components, heritability and genetic gains based on pedigree information are shown in Table 2. The heritability estimates varied from 0.04 to 0.46. Wood density (WD) was the most heritable trait ($h_{a}^{2}$ = 0.46), while diameter at breast height (DBH), stem straightness (ST), and branch quality (BQ) had the lowest heritability estimates in this breeding population ($h_{a}^{2}$ = 0.04, 0.06, and 0.05, respectively). Additionally, the estimated genetic gains based on pedigree information were 7.7%, 2.9%, 4.4%, 3.9% and 9.7% for tree height (HT), DBH, ST, BQ and WD, respectively, considering an intensity of selection of ~10% (10.06%; *n* = 65).

### 2.3. Prediction Based on Genomic Data

**Figure 1:**
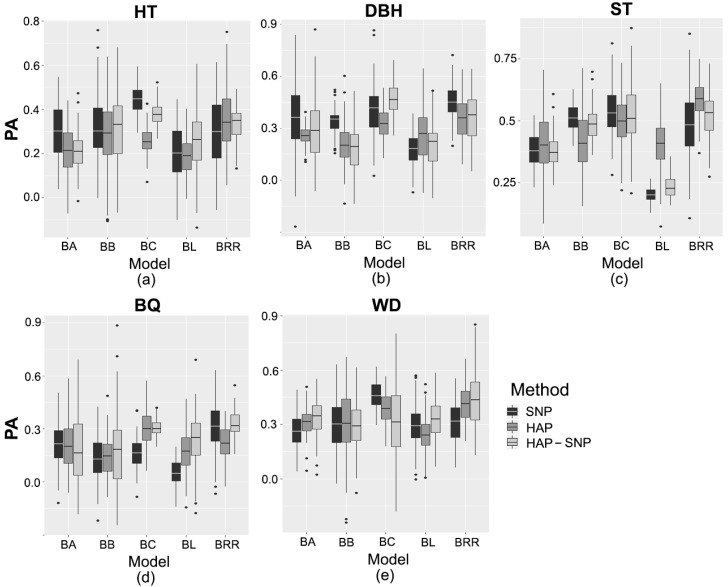
Predictive ability (PA) of (**a**) tree height (HT), (**b**) diameter at breast height (DBH), (**c**) stem straightness (ST), (**d**) branch quality (BQ), and (**e**) wood density (WD). Models based on SNP markers (SNP), haplotypes (HAP), and haplotypes with SNPs that were not assigned to a haplotype (HAP-SNP) are represented by black, dark gray and light gray bars, respectively. BA, BB, BC, BL, and BRR correspond to Bayes A, Bayes B, Bayes C, Bayesian Least Absolute Shrinkage and Selection Operator, and Bayesian Ridge Regression, respectively. Each box-plot represents the distribution of PA values for 100 cycles of cross-validation.

The genomic prediction methods were compared using the average values of marginal posterior distributions of each estimated parameter. Genomic heritability, genetic gains and predictive ability (PA) values, obtained for each trait and prediction model, are shown in Table 2 and Table 3 and Figure 1. PA values were dependent on the genomic prediction models and marker type (SNP markers, haplotypes (HAP) and haplotypes in conjunction with SNPs that were not assigned to a haplotype (HAP-SNP)). PA values for HT varied between 0.19 and 0.44. For Bayes B (BB) and Bayesian Ridge Regression (BRR) methods, the PA values based on SNP, HAP and HAP-SNP were not statistically different from each other, but Bayes A (BA), Bayesian Least Absolute Shrinkage and Selection Operator (Bayesian LASSO or BL) and Bayes C (BC) methods based on the three markers (SNP, HAP and HAP-SNP) showed significant differences in terms of PA values. Based on the comparison among the assessed models, BC had the highest predictive ability (PA = 0.44 (SNP) and 0.38 (HAP-SNP)) for HT in most cases, and this method consistently gave one of the highest values for genomic heritability (${\hat{h}}_{g}^{2}$ = 0.36) and genetic gain (GG = 8%). In addition, the genomic heritability based on this method (either SNP or HAP-SNP) was statistically higher than the pedigree-based heritability (${\hat{h}}_{a}^{2}$ = 0.15 [0.01–0.28]). On the other hand, the genetic gains for HT based on pedigree information and BC methods were not statistically different.

For DBH, PA values ranged between 0.17 and 0.46. The PA varied significantly among models based on SNP, HAP and HAP-SNP. In general, the models based on SNP had the highest PA values. Among the models based on SNP, BRR had the highest predictive ability for DBH (PA = 0.45), while HAP-SNP-based BC had the highest predictive ability (PA = 0.46). The genomic heritability estimates based on BC and BRR ranged between 0.26–0.32 and 0.12–0.16, respectively, and were significantly higher than the pedigree-based heritability (${\hat{h}}_{a}^{2}$ = 0.04 [<0.01–0.10]). Consistently, the genetic gains based on the selection with these genomic models were statistically higher than those based on pedigree information (GG = 2.9% [1.4–4.5]).

The predictive ability of ST varied from 0.20 to 0.58. On the other hand, the PA values of ST were not statistically different among markers (SNP, HAP or HAP-SNP) in most cases. BB and BC had the highest PA among the models based on SNP (PA = 0.52 and 0.54, respectively) and HAP-SNP (PA = 0.52 for BB and BC), while BRR had the highest predictive ability (PA = 0.58) among the HAP-based models. Consistently, all genomic heritability estimates based on BB, BC and BRR (${\hat{h}}_{g}^{2}$ = 0.15–0.34) were higher than the pedigree-based heritability (${\hat{h}}_{a}^{2}$ = 0.06 [<0.01–0.14]). In addition, only BC and BRR had genetic gain estimates statistically higher than those based on pedigree information for ST.

The predictive ability of BQ ranged between 0.06 and 0.33, which did not vary significantly between models based on SNP, HAP or HAP-SNP in most cases. Among the models based on SNP, BC had the highest predictive ability of BQ (PA = 0.31). For the HAP-based models, the highest PA was obtained by BC (PA = 0.28). In accordance with this, BC and BRR had the highest PA of BQ among the HAP-SNP-based models (PA = 0.31 and 0.33, respectively). All genomic heritability estimates based on BC and BRR (${\hat{h}}_{g}^{2}$ = 0.12–0.29) were higher than the pedigree-based heritability estimate (${\hat{h}}_{a}^{2}$ = 0.05 [<0.01–0.11]). However, only BRR had genetic gain estimates statistically higher than those based on pedigree information.

The predictive ability of WD ranged between 0.24 and 0.46, which varied significantly between models based on SNP, HAP and HAP-SNP in most cases. Among SNP-based models, BC had the highest predictive ability of WD (PA = 0.46). For HAP-based models, the highest predictive ability was obtained by BC and BRR (PA = 0.41 and 0.39, respectively). In addition, for HAP-SNP-based models, BRR had the highest predictive ability (PA = 0.44). The heritability of WD based on any genomic model (${\hat{h}}_{g}^{2}$ = 0.04–0.26) was statistically lower than the pedigree-based heritability (${\hat{h}}_{a}^{2}$ = 0.46 [0.22–0.69]). Consistently, the genetic gains for WD based on any genomic prediction model did not exceed 5% and were statistically lower than those based on pedigree information. 

**Figure 2:**
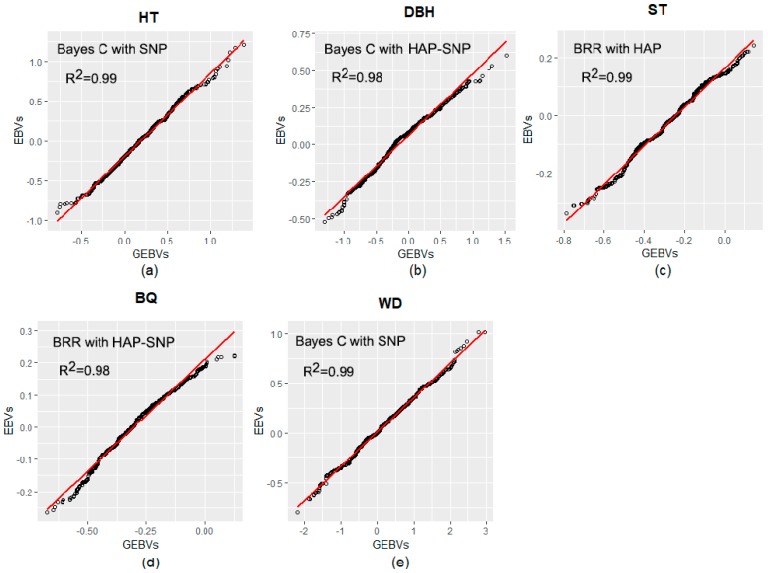
Linear regression plots relating estimated breeding values (pedigree-based Estimated Breeding Values; EBVs) and genomic-based EBVs (GEBVs). (**a**) EBVs and GEBVs for tree height (HT); (**b**) EBVs and GEBVs for diameter at breast height (DBH); (**c**) EBVs and GEBVs for stem straightness (ST); (**d**) EBVs and GEBVs for branch quality (BQ); and (**e**) EBVs and GEBVs for wood density (WD).

The regressions between pedigree-based Estimated Breeding Values (EBVs) and genomic-based EBVs (GEBVs considering models with the highest predictive ability for each trait) are shown in Figure 2. All coefficients of determination (R^2^) between EBVs and GEBVs for all traits were above 0.98.

## 3. Discussion

### 3.1. Haplotype Blocks Construction

Previously, Durán et al. [45], Thavamanikumar et al. [46] and Cappa et al. [47] determined that the linkage disequilibrium (LD) in natural and controlled populations of *E. globulus* decreases rapidly in the range of 1000–4000 bp. However, in this study, several genomic regions were detected in strong disequilibrium above 10,000 bp. About 24% and 2% of the haplotype blocks formed had an extension over 10,000 bp and 100,000 bp, respectively, with a disequilibrium coefficient (D′) value higher than 0.7. The construction of the haplotype blocks was based on the confidence interval algorithm of Gabriel et al. [48], which establishes that those pairs of SNPs that are in a strong linkage disequilibrium have a D′ value between 0.7 and 0.98, considering a confidence interval of 95%. These D′ values could reveal that, historically, the probability of recombination between both loci is quite low. In this study, more than 1300 haplotype blocks that would meet this condition were detected. Therefore, it is possible to assume that the SNPs grouped in blocks are in strong LD and they are possibly inherited together across generations.

In breeding populations of self-pollinated plants and controlled crosses, it is expected that LD decays over long distances. For example, in soybean and rice, LD can be significant at a distance of 100,000 bp and 250,000 bp between loci, respectively [49], while in the genomes of outcrossing plants, LD is expected to decay at short distances due to the reproductive mechanisms that underlie these species, and their loci tend to be heterozygous [50,51]. In the context of this study, the genotyped individuals were sampled from a breeding population formed by full- and half-sib families, which could increase the probability of the occurrence of regions that form haplotype blocks.

### 3.2. Performance of Pedigree and Genomic Prediction Models

According to the pedigree and genomic prediction models, the traits studied in this population were found to be weakly to highly heritable. The results indicated that DBH, ST and BQ are under relatively low genetic control. However, previous studies have reported that the heritability of DBH in *Eucalyptus* spp. can be greater than 0.1 in populations from 3 to 15 years of age [52,53,54,55]. For DBH, the genomic prediction models based on BC and BRR had higher heritability values than those based on pedigree, with a genetic gain up to ~8%. Previously, Tan et al. [56] determined that the genomic heritability of DBH, based on Ridge Regression Best Linear Unbiased Prediction (BLUP) and Reproducing Kernel Hilbert Spaces, was higher than the pedigree-based heritability estimate in *Eucalyptus* hybrids at 6 years of age. In this study, SNP-based BRR and HAP-SNP-based BC had the highest predictive ability for DBH, but the genomic heritability estimated by BC (HAP-SNP) was two times higher than the BRR model based on SNP markers. In BC, the prior assumptions of all SNP effects have a common variance, and the method assigns a nonnull prior probability for the marker effect to be equal to zero [5,57]. Due to this assumption, Bayes C has been used to identify QTLs with large effects [58]. Similarly, BRR assumes a common variance for all SNP markers, but all SNP effects are shrunk to a similar extent [7]. In contrast, Suontama et al. [59] and Resende et al. [6] reported that the genomic heritability of DBH was not superior to the heritability estimated by BLUP in *Eucalyptus nitens* and hybrids of *Eucalyptus urophylla* × *E. grandis*, respectively. In another study, Müller et al. [60] reported that the genomic heritability of DBH was lower than the estimated heritability by BLUP in *E. benthamii*. Interestingly, Müller et al. [60] performed a prediction for DBH using 13,787 and 10,460 SNPs that were not in LD, which could confirm our hypothesis that the inclusion of SNPs in strong LD (such as a BC model based on HAP-SNP) could be beneficial for genetic parameter estimation.

The heritability of ST has been previously reported as low [54,61], which is consistent with the pedigree-based model and some genomic prediction models assessed in the present study. However, other studies have found that the heritability of ST in *E. globulus* can be moderate [62] and even high (${\hat{h}}_{a}^{2}$ > 0.3, [53]). Almost all genomic-based heritability estimates of ST were statistically higher than those estimated by the pedigree-based method. Suontama et al. [59] found that the pedigree-based heritability of stem straightness in *E. nitens* increased approximately twice when using marker-based models. In the context of our study, the highest predictive ability for ST was obtained by BC based on SNP, BRR based on HAP, and BRR and BC models based on HAP-SNP. BC and BRR models had higher heritability than the estimate based on pedigree. However, BC models (based on either SNP or HAP) had a higher genomic heritability than other prediction models. There have been conflicting reports on the genetic architecture of stem straightness in trees. For example, Bartholomé et al. [63] detected QTLs that explained up to 5% of the total phenotypic variation of ST in maritime pine. Yang et al. [64] found QTLs that explained up to 15% of the total phenotypic variation of the stem straightness of Pinus hybrids. Additionally, Arriagada et al. [65] reported five QTLs for ST, explaining a total of 6–14% of the total proportion of trait variation in *Eucalyptus cladocalyx*.

BQ has been a scarcely evaluated trait in breeding programs of *E. globulus*. For instance, Callister et al. [53] reported a range in branching quality (measured as branch thickness) of 0–0.16 in full-sib families of E. globulus (at age 3.5). Ballesta et al. [43] reported that the pedigree-based heritability estimate of BQ in *E. globulus* at 4 years of age was less than 0.1. In other tree species, traits related to branch quality have been described to be moderately heritable [66,67]. In the present study, among the models based on SNP and HAP markers, BC had the highest predictive ability of branch quality. On the other hand, BC and BRR had the highest PA values among the models based on HAP-SNP. Interestingly, all genomic heritability estimates based on BC and BRR were higher than the pedigree-based heritability estimates. However, this result should be interpreted with caution due to Bayes C performing variable selection and shrinkage procedures, which could mean that the polygenic background may not be taken into account, favoring a selection based on major effect genes [68]. This could explain why the genetic gains based on BC were lower than those based on BRR.

Several studies have shown that WD is a highly heritable trait in *Eucalyptus* spp. [59,62,69], which is consistent with the estimates from the pedigree-based model and BC based on SNP, HAP and HAP-SNP. None of the methods based on genomic data exhibited higher heritability (and genetic gain) for this trait than the pedigree-based method. In other studies, Suontama et al. [59] reported that genomic-based and pedigree-based heritability values were similar for the basic wood density in *E. nitens*. Consistent with our findings, Resende et al. [6] reported that the genomic heritability of WD is subtly lower than the pedigree-based heritability in hybrids of *E. urophylla* and *E. grandis*. In other tree species, Beaulieu et al. [70] reported that the heritability of the wood density in trees of *Picea glauca* is lower when estimated by genomics than pedigree. As expected, the highest value of genetic gain was obtained for WD, while the lowest value was obtained for DBH. In this sense, the selection based on genomics for traits such as DBH, ST and BQ is highly justified and particularly attractive because of the potential of enhancing selection accuracy for low heritability traits and increasing the genetic gains for these traits [6,71]. On the other hand, Beaulieu et al. [70] stated that one of the reasons why genomic selection might not be as effective for predicting wood density is that pedigree information makes it possible to capture loci that have not been considered in a genomic prediction model, which can be important for the genetic control of the trait. According to our results, the BC model had a higher predictive ability and genomic heritability in most cases, supporting the idea of an oligogenic architecture for WD. Contrarily, Durán et al. [45] reported that BC model had a lower PA than the BL model.

The genomic heritability for HT, estimated by a BC model (SNP and HAP-SNP), was higher than the pedigree-based estimate. However, the HAP-SNP-based BC model had a predictive ability lower than the model based on SNP markers. In accordance with this, Tan et al. [56] determined that the genomic heritability of HT, based on Ridge Regression BLUP and Reproducing Kernel Hilbert Spaces, was superior to the pedigree-based heritability in *Eucalyptus* hybrids. However, the genetic gains estimated using the pedigree and BC methods were not statistically different. Previously, Lenz et al. [72] and Beaulieu et al. [70] reported that the genetic gains based on genomic prediction could be lower than the gains based on the pedigree method for HT in other tree species. As mentioned, the BC model (based on SNP and HAP-SNP) had the highest predictive ability for HT in the most cases. Contrarily, Müller et al. [60] found no differences in the predictive ability of growth traits (HT and DBH) between the BA, BB, BC, BL, and BRR models.

Several studies in animals have emphasized the use of haplotypes (from high-density DNA arrays) to estimate the predictive ability of different GS models [27,35,36,37,38]. In plants, Matias et al. [29] demonstrated that the use of haplotypes in prediction studies in maize increased its predictive ability by 20%. It should be noted that in outcrossing plants, such as *Eucalyptus* and other forest species, LD usually decays at short genomic distances, which allows the identification of smaller haplotype blocks and with fewer variants (haplotypes). In this study, we found haplotype blocks formed by SNPs in a strong LD (D′ > 0.7) with a size above 300 kbp. According to Cuyabano et al. [27], haplotypes that are constructed by SNPs in a LD of D′ > 0.45 can significantly increase the predictive ability of a genomic selection model. Our results revealed that predictive ability values were mainly dependent on the Bayesian methods assessed (i.e., BA, BB, BC, BL and BRR) more than on the marker type (SNP, HAP or HAP-SNP). However, genomic models that included the haplotype effect (either HAP or HAP-SNP) significantly increased the PA of low-heritability traits. These results, nevertheless, should be interpreted with caution due to the age of the trees, and we therefore emphasize that further studies are needed to evaluate the performance of the genomic models. On the other hand, the development of techniques to select trees at early growth stages may greatly increase the genetic gain per unit time, and thus, substantially accelerate tree-breeding programs.

## 4. Materials and Methods

### 4.1. Trial Conditions and Phenotyping 

In 2018, 6-year-old trees of *Eucalyptus globulus* from a progeny trial consisting of a mix of half-sib and full-sib families located in the La Poza sector, Purranque, Chile [43], were evaluated according to the following traits (description in Table S1 and Figure S2): wood density (WD), stem straightness (ST), branch quality (BQ), diameter at breast height (DBH), and tree height (HT). This location (40°57′S, 73°30′W; 326 m.a.s.l.) has an Oceanic or Marine climate type with an annual accumulated rainfall of 1282 mm and an average annual temperature of 13 °C. The WD was measured indirectly according to Valenzuela et al. [69]. ST was evaluated in the first 2/3 of the total height of the tree according to an ordinal scale (seven levels). The value 0 corresponds to trees that have a curvature in the first third of the total height of the tree and 6 in the case of trees that could present a slight curvature in the upper third of the total height of the tree without affecting productivity. BQ was evaluated according to different criteria that define quality (diameter, angle and distribution of branches in the tree) by means of an ordinal scale of six levels, in which a value of 0 is assigned to trees with an extreme deficiency in the diameter of branches and any other variable, and a value of 6 corresponds to trees that have an optimal combination of all quality variables without generating loss of productivity. The trees were distributed in a randomized complete block design with 30 blocks, considering single-tree plots (each family is represented by only one tree in each block) and a planting density of 2.5 m between each tree within each block.

### 4.2. Genotyping, Linkage Disequilibrium and Haplotype Blocks

Genomic DNA was isolated from the leaves of 646 randomly selected individuals of *E. globulus* (~10 individuals per family). The DNA extraction protocol followed the work of Ballesta et al. [43]. Individuals were genotyped using the EUChip60K SNP system (GeneSeek, Lincoln, NE, USA) [17]. The genotyping quality of the samples was evaluated in Genome Studio software (Illumina, San Diego, CA). Monomorphic SNP markers and those with a call rate <90% were removed. Subsequently, those SNPs with a minor allele frequency (MAF) <0.05 were eliminated. A total of 14,442 remaining SNPs was retained for the 646 individuals.

The haplotype blocks were defined according to the confidence interval algorithm developed by Gabriel et al. [48] using the software Haploview v. 4.2 [73]. The pairs of SNPs were considered to be in strong linkage disequilibrium (LD) if the upper limit of the 95% confidence interval of the value of normalized disequilibrium coefficient (D′) was higher than 0.98 and if the lower limit had a minimum value of 0.7. The D′ between A and B loci, was calculated as follows:

(1) $${D^{\prime }}_{\mathrm{AB}}=D/D_{\mathrm{MAX}}$$

where D is calculated as $D=p_{A_{1}B_{1}}p_{A_{2}B_{2}}-p_{A_{1}B_{2}}p_{A_{2}B_{1}}$, and $D_{\mathrm{MAX}}$:

(2) $$D_{\mathrm{MAX}}=\{\begin{array}{c} -\min\{p_{A_{1}}p_{B_{1}}{,p}_{A_{2}}p_{B_{2}}\},\;\mathrm{when}\;D<0\\ \min\{p_{A_{1}}p_{B_{2}}{,p}_{A_{2}}p_{B_{1}}\},\;\mathrm{when}\;D\geq 0 \end{array}$$

The physical positions of each SNP were established according to the consensus map of the genome of *E. grandis* [74]. The extent of LD was also estimated as the squared allele frequency correlation (*r*^2^). The critical *r*^2^ value was calculated according to the method used by Breseghello and Sorells [75].

### 4.3. Prediction Models Based on Pedigree and Genomic Data

In this study, prediction models based on pedigree and genomic data from an array of SNP markers were used. In the pedigree-based model, individual breeding values were predicted using a Bayesian generalized linear model implemented in the MCMCglmm (Markov Chain Monte Carlo—Generalized Linear Mixed Model) library [76] of R 3.6.1 [77] This Bayesian analysis was carried out using the following base model:

(3) y=Xβ+Za+ε

where y corresponds to the phenotypic data vector, β is the vector of block effects, a is the vector of the additive genetic effects $a\sim N\left(0,A\sigma_{a}^{2}\right)$, A corresponds to the matrix of Wright’s coefficients (pedigree information), and $\sigma_{a}^{2}$ is the additive genetic variance. X and Z correspond to the known incidence matrices that relate the observation vector (y) to vectors β and a, respectively, and ε corresponds to the vector of residual effects, $\varepsilon \sim N\left(0,I\sigma_{\varepsilon }^{2}\right)$, where I is an identity matrix, and $\sigma_{\varepsilon }^{2}$ is the residual variance. The Bayesian models were run with 1,000,000 iterations, a burn-in period of 100,000 and a thin of 50.

The prediction models based on SNPs/haplotypes were the following: Bayesian Least Absolute Shrinkage and Selection Operator (Bayesian LASSO or BL, [78,79]), Bayesian Ridge Regression (BRR, [7]), Bayes A (BA, [1]), Bayes B (BB, [1]) and Bayes Cπ (BC, [5]). All whole-regression models can be expressed in matrix form as follows:

(4) y=Xβ+Zm+ε

where y corresponds to the phenotypic data vector, β is the vector of block effects, m corresponds to marker effects (SNPs and/or haplotypes), and depending on the model, different prior distributions are assigned; for instance, a double exponential distribution in the case of BL and a Gaussian distribution in the case of BRR, among others (see Refs. [7,57]). ε corresponds to the vector of residuals, $\varepsilon \sim N\left(0,I\sigma_{\varepsilon }^{2}\right)$. X and Z correspond to the incidence matrices that relate the observation vector (y) to vectors β and m, respectively.

The matrix of SNP markers or haplotypes was coded by the numbers 0, 1 and 2. In the case of SNP markers, 0 represents the homozygous genotype of the allele with the lowest frequency for the *i*-th marker (*i* = 1, …, n), 1 represents the heterozygous genotype for the *i*-th marker, and 2 represents the homozygous genotype of the allele with the highest frequency for the *i*-th marker. In the case of haplotypes, since one haplotype block can have more than two allelic variants, the values of 0, 1 and 2 represent the number of copies for each variant (haplotype), in which a value of 0 was assigned for those individuals who did not present any copy of the *j*-th haplotype and *i*-th block, a value of 1 was assigned if they presented a copy of the *j*-th haplotype and *i*-th block, and a value of 2 was assigned if they presented two copies of the *j*-th haplotype and *i*-th block [27]. The models based on SNP markers, haplotypes and haplotypes in conjunction with SNPs (that were not assigned to a haplotype) are identified by SNP, HAP and HAP-SNP, respectively.

The BL method assumes that the marker effects are distributed a priori according to a double exponential (DE), $p\left(m_{i}{|\lambda ,\sigma }_{\varepsilon }^{2}\right)=\mathrm{DE}\left(m_{i}{|0,\lambda ,\sigma }_{\varepsilon }^{2}\right)$, where λ corresponds to a regularization parameter. The distribution of DE generates a strong contraction (close to zero) to estimate the effects of the markers. BRR is a Bayesian method based on the fact that model regressors (SNPs and/or haplotypes) have a common variance ($\sigma_{m}^{2}$); those regressors with the same allelic frequency explain the same proportion of the additive variance and have the same contraction effect [7]. The marker effect ($m_{i}$) is distributed as follows: $m_{i}|\sigma_{m}^{2}\sim N\left(0,\sigma_{m}^{2}\right)$; and the common variance $p(\sigma_{m}^{2})$~scaled inverse Chi-squared ($\sigma_{m}^{2}|\upsilon_{m},S_{m}\sim \chi^{-2}\left(\upsilon_{m},S_{m}\right)$), with degree of freedom and scale parameters $\upsilon_{m}$ and $S_{m}$, respectively. In BA, the marginal distribution of marker effects is a scaled-t density, in which, for computational convenience, this density is implemented as an infinite mixture of scaled-normal densities (see Ref. [57]). The variance of each marker is assumed to be distributed scaled inverse Chi-squared. BB uses a mixed distribution with a mass at zero, such that the prior distribution of the effects of the all markers is given by [51]

(5) $$m_{i}|\sigma_{m_{i}}^{2},\pi =\{\begin{array}{c} 0\;\mathrm{with}\;\mathrm{probability}\;\pi \\ N\left(0,\sigma_{m_{i}}^{2}\right)\;\mathrm{with}\;\mathrm{probability}\;1-\pi \end{array}$$

A scaled inverse Chi-square prior distribution $\chi^{-2}\left(\upsilon_{m},S_{m}\right)$ is assumed for $\sigma_{m_{i}}^{2}$ (*i* = 1, …, n), which is equal for all markers. In BC, all markers are considered to have a common variance ($\sigma_{m}^{2}$) and promote the selection of variables such as Bayes B. The marker effects are assumed to be $m_{i}|\sigma_{m_{i}}^{2}\sim N\left(0,\sigma_{m}^{2}\right)$ with a probability of 1 − π = 0.

All the genomic-based Bayesian methods were implemented in the library BGLR (Bayesian Generalized Linear Regression) [57] of R 3.6.1 [77]. Variance components were estimated with a total of 1,000,000 iterations, a burn-in period of 100,000, and a thin of 50. The predictive ability (PA) of each model was measured as the correlation between the Genomic Estimated Breeding Values (GEBVs) obtained by Equation (2) and genomic breeding values predicted by cross-validation, which considered 90% of the individuals as the training population and the remaining 10% as the validation population. The PA was reported as the average of correlation coefficients for 100 cycles of the cross-validation.

### 4.4. Heritability and Genetic Gain

The prediction methods (genomic and pedigree-based) were also compared by the values of heritability and genetic gains. In the case of the pedigree-based method [80], the heritability (${\hat{h}}_{a}^{2}$) in a narrow sense was calculated as follows:

(6) $${\hat{h}}_{a}^{2}=\frac{{\hat{\sigma }}_{a}^{2}}{{\hat{\sigma }}_{a}^{2}+{\hat{\sigma }}_{\varepsilon }^{2}}$$

where ${\hat{\sigma }}_{a}^{2}$ and ${\hat{\sigma }}_{\varepsilon }^{2}$ correspond to the additive genetic and residual variances, respectively. In the case of Bayesian genomic prediction models (SNP/haplotypes), genomic heritability (${\hat{h}}_{g}^{2}$), genomic variance (${\hat{\sigma }}_{g}^{2}$) and the residual variance (${\hat{\sigma }}_{\varepsilon }^{2}$) were estimated using the marginal posterior distributions of each estimated parameter [81,82,83]. The genomic variance was estimated for each model as follows:

For BRR and BC:

(7) $${\hat{\sigma }}_{g}^{2}=2\;{\hat{\sigma }}_{m}^{2}\sum_{i=1}^{n}p_{i}(1-p_{i})$$

For BA and BB:

(8) $${\hat{\sigma }}_{g}^{2}=2\sum_{i=1}^{n}p_{i}(1-p_{i}){\hat{\sigma }}_{m_{i}}^{2}$$

For BL:

(9) $${\hat{\sigma }}_{g}^{2}=2\sum_{i=1}^{n}\tau_{i}^{2}{\hat{\sigma }}_{\varepsilon }^{2}p_{i}(1-p_{i})$$

where $p_{i}$ is the MAF of *i*th marker, ${\hat{\sigma }}_{m}^{2}$ is the variance of markers, and $\tau_{i}^{2}$ was assumed to be the exponential of λ, $\tau_{i}^{2}\sim \mathrm{Exp}\left(\lambda^{2}\right)$, in which λ was assumed to belong to Gamma distribution $\lambda^{2}\sim G\left(\varphi_{1}{,\varphi }_{2}\right)$. BRR and BC models assume that all markers have the same variance (${\hat{\sigma }}_{m}^{2}$), while BB and BA models assume a variance for each *i*th marker (${\hat{\sigma }}_{m_{i}}^{2}$). BB, BC and BL models assume the selection of variables; however, BL uses regularization parameter λ that directs markers with irrelevant effects close to zero.

The genetic gain (GG) was estimated for each prediction method using the following expression [54,84]:

(10) $$\mathrm{GG}=\frac{\left({\bar{y}}_{\mathrm{sel}}-{\bar{y}}_{\mathrm{pop}}\right)}{{\bar{y}}_{\mathrm{phe}}}*100$$

where ${\bar{y}}_{\mathrm{sel}}$ and ${\bar{y}}_{\mathrm{pop}}$ correspond to the estimated posterior mean of the breeding values of selected trees (with a selection intensity of ~ 10.06%) and the estimated posterior population mean of the breeding values, respectively, and ${\bar{y}}_{\mathrm{phe}}$ is the phenotypic mean for each trait. For ordinal traits, the ${\bar{y}}_{\mathrm{phe}}$ term was calculated according to Burdon et al. [85].

## 5. Conclusions

To our knowledge, this study is one of the first to examine the inclusion of haplotypes in genomic selection models of *Eucalyptus*. In general, genomic heritability estimates were higher than those based on pedigree information for most of the studied traits. On the other hand, the predictive ability values were dependent on the genomic prediction models and marker type. On average, the homocedastic methods (BRR and Bayes C) had the highest predictive ability for the majority of traits. Notably, genomic models that included the haplotype effect (either HAP or HAP-SNP) significantly increased the PA of traits with low heritability. The results of this study provide additional perspectives for the implementation of genomic selection in *Eucalyptus* breeding programs, which could be especially beneficial for improving low heritability traits.

## Figures and Tables

**Table 1:** Summary of information on haplotypes and haplotype blocks determined in a breeding population of *E. globulus*. Ch corresponds to the chromosome number and single nucleotide polymorphic markers (SNPs) to the number of SNPs detected; HAP-Blocks is the number of haplotype blocks constructed; HAPs is the number of haplotypes; Max (kbp) corresponds to the maximum size (in kbp) for the haplotype blocks; Min (bp) corresponds to the minimum size (in bp) for the haplotype blocks; and Min (SNPs) and Max (SNPs) correspond to the maximum and minimum number of SNPs forming the haplotype blocks, respectively.

Ch	SNPs	HAP-Blocks	HAPs	Max (kb)	Min (bp)	Max (SNPs)	Min (SNPs)
1	924	75	219	381	61	6	2
2	1766	121	370	357	36	6	2
3	1587	99	299	123	30	11	2
4	893	71	207	31	31	5	2
5	1500	83	238	279	49	8	2
6	1474	144	407	343	63	6	2
7	1220	87	248	356	121	5	2
8	1811	152	418	482	70	6	2
9	946	89	249	94	34	5	2
10	1065	103	295	318	49	10	2
11	1236	113	329	250	75	12	2
Total	14,422	1137	3279	-	-	-	-
Mean	1311	103	298	274	54	7	2

**Table 2:** Estimates of pedigree-based heritability (${\hat{h}}_{a}^{2}$), genomic heritability (${\hat{h}}_{g}^{2}$) and genetic gain (GG; percentage) for each method of prediction based on pedigree (PBP), SNP markers (SNP), haplotype (HAP), and haplotypes and SNPs that were not assigned to a haplotype (HAP-SNP). BA, BB, BC, BL, and BRR correspond to Bayes A, Bayes B, Bayes C, Bayesian Least Absolute Shrinkage, and Selection Operator and Bayesian Ridge Regression, respectively.

Trait/Model	Pedigree		SNP		HAP		HAP-SNP	
	${\hat{h}}_{a}^{2}$ [CR]	GG [CR]	${\hat{h}}_{g}^{2}$	GG	${\hat{h}}_{g}^{2}$	GG	${\hat{h}}_{g}^{2}$	GG
Tree height								
PBP	0.15 [0.01–0.28]	7.7 [5.4–10.3]	-	-	-	-	-	-
BA	-	-	0.11	5.6	0.06	4.2 *	0.10	5.2 *
BB	-	-	0.27	6.0	0.11	3.6 *	0.29 *	6.3
BC	-	-	0.36 *	7.9	0.28	6.6	0.36 *	7.8
BL	-	-	0.07	4.2 *	0.04	3.1 *	0.06	3.6 *
BRR	-	-	0.19	8.7	0.14	7.6	0.20	8.6
Diameter at breast height								
PBP	0.04 [<0.01–0.10]	2.9 [1.4–4.5]	-	-	-	-	-	-
BA	-	-	0.08	5.0 *	0.04	3.8	0.07	4.2
BB	-	-	0.19 *	4.9 *	0.09	3.4	0.14 *	3.3
BC	-	-	0.31 *	7.2 *	0.26 *	6.6 *	0.32 *	7.2 *
BL	-	-	0.05	3.6	0.05	4.1	0.05	3.4
BRR	-	-	0.16 *	8.3 *	0.12 *	7.8 *	0.16 *	8.2 *
Stem straightness								
PBP	0.06 [<0.01–0.14]	4.4 [1.9–7.1]	-	-	-	-	-	-
BA	-	-	0.10	4.4	0.09	4.7	0.09	4.1
BB	-	-	0.26 *	5.7	0.18 *	4.7	0.28 *	5.5
BC	-	-	0.34 *	7.1	0.30 *	7.5 *	0.34 *	7.2 *
BL	-	-	0.05	2.7	0.04	2.8	0.07	3.2
BRR	-	-	0.18 *	7.6 *	0.15 *	7.9 *	0.20 *	8.0 *
Branch quality								
PBP	0.05 [<0.01–0.11]	3.9 [1.4–6.0]	-	-	-	-	-	-
BA	-	-	0.04	2.2	0.04	2.7	0.05	2.3
BB	-	-	0.12 *	2.4	0.10	2.7	0.08	1.8
BC	-	-	0.29 *	5.0	0.25 *	5.4	0.29 *	4.9
BL	-	-	0.03	2.0	0.03	2.3	0.04	2.0
BRR	-	-	0.15 *	6.2 *	0.12 *	6.4 *	0.15 *	6.1 *
Wood density								
PBP	0.46 [0.22–0.69]	9.7 [7.5–12]	-	-	-	-	-	-
BA	-	-	0.07 *	2.0 *	0.05 *	2.0 *	0.08 *	2.1 *
BB	-	-	0.17 *	2.2 *	0.12 *	1.9 *	0.16 *	2.1 *
BC	-	-	0.34	3.2 *	0.26	3.0 *	0.33	3.1 *
BL	-	-	0.06 *	1.7 *	0.04 *	1.7 *	0.06 *	1.8 *
BRR	-	-	0.16 *	3.6 *	0.12 *	3.4 *	0.17 *	3.5 *

Numbers with asterisks are statically different from pedigree-based estimates (90% Bayesian credible sets). CR: 90% credible region from marginal posterior distributions.

**Table 3:** Estimates of predictive ability (average of 100 cross-validation cycles) of Bayesian models based on SNPs (SNP), haplotypes (HAP) and haplotypes with SNPs that were not assigned to a haplotype (HAP-SNP) for each studied trait.

Trait/Markers	Genomic Model				
	BA	BB	BC	BL	BRR
Tree height					
SNP	0.31 bA	0.32 bA	**0.44** aA	0.21 cB	0.30 bA
HAP	0.21 cdB	0.28 bA	0.25 bcC	0.19 dB	0.35 aA
HAP-SNP	0.21 dB	0.31 bA	0.38 aB	0.26 cA	0.33 bA
Diameter at breast height					
SNP	0.35 bA	0.34 bA	0.39 bB	0.17 cB	0.45 aA
HAP	0.26 bcB	0.21 cB	0.33 aC	0.26 bA	0.36 aB
HAP-SNP	0.28 cAB	0.19 dB	**0.46** aA	0.20 dB	0.37 bB
Stem straightness					
SNP	0.38 cA	0.52 abA	0.54 aA	0.20 dC	0.48 bB
HAP	0.40 cA	0.42 cB	0.50 bA	0.40 cA	**0.58** aA
HAP-SNP	0.38 bA	0.49 aA	0.52 aA	0.23 cB	0.52 aB
Branch quality					
SNP	0.22 bA	0.13 cA	0.16 cB	0.06 dC	0.31 aA
HAP	0.20 bA	0.14 cA	0.28 aA	0.17 bcB	0.22 bB
HAP-SNP	0.19 bA	0.18 bA	0.31 aA	0.24 bA	0.33 aA
Wood density					
SNP	0.26 bB	0.30 bA	**0.46** aA	0.29 bA	0.32 bB
HAP	0.32 bA	0.31 bA	0.39 aB	0.24 cB	0.41 aA
HAP-SNP	0.34 bA	0.29 bA	0.32 bC	0.33 bA	0.44 aA

BA: Bayes A; BB: Bayes B; BC: Bayes C; BL: Bayesian Least Absolute Shrinkage and Selection Operator; BRR: Bayesian Ridge Regression. Statistical significance between different genomic models (BA, BB, BC, BL and BRR) is noted by lowercase letters, while that between different markers (SNP, HAP and HAP-SNP) is shown by upper case letters. Different letters show the statistical significance at *p* < 0.01 using the Tukey–Kramer test. Numbers in bold show the highest PA estimates considering both approaches: genomic models and marker types (SNP, HAP or HAP-SNP).

## Supplementary Materials

The following are available online at https://www.mdpi.com/2223-7747/8/9/331/s1, Figure S1: Genome-wide average linkage disequilibrium (LD) decay plot estimated across all chromosomes of *Eucalyptus* for the studied population. The LD values correspond to the average of the correlation between alleles at two loci (*r*^2^) and the normalized disequilibrium coefficient (D′) for each 1 Kpb. The LD threshold of *r*^2^ = 0.14 is indicated with a red line; Figure S2: Distributions and histograms of the studied traits: (a) tree height (HT) in m, (b) diameter at breast height (DBH) in cm, (c) stem straightness (ST), (d) branch quality (BQ) and (e) wood density (WD) in mm; Table S1: Summary of phenotypic information for quantitative traits related to wood quality and tree growth measured in a six-year-old breeding population of *E. globulus*.
