# Supplementary material for: SNP and Haplotype-Based Genomic Selection of Quantitative Traits in Eucalyptus globulus
Source: Plants (Basel). 2019 Sep 5;8(9):331. doi: 10.3390/plants8090331 (PMC6783840; doi:10.3390/plants8090331)
Supplement: Supplementary file 1 [file plants-08-00331-s001.pdf]

Supplementary Material

SNP and haplotype-based genomic selection of quantitative traits in *Eucalyptus globulus*

Paulina Ballesta [1], Carlos Maldonado [1], Paulino Pérez-Rodríguez[2] and Freddy Mora[1,*]

[1] Institute of Biological Sciences, University of Talca, 2 Norte 685, Talca 3460000, Chile; pballesta@utalca.cl (P.B.); camaldonado@utalca.cl (C.M.); fmora@utalca.cl (F.M.).

[2] Colegio de Postgraduados, Statistics and Computer Sciences, Montecillos, Edo. de México, México; perpdgo@colpos.mx (P.P).

* Correspondence: fmora@utalca.cl; Tel.: +56-71-2200280.

Received: date; Accepted: date; Published: date

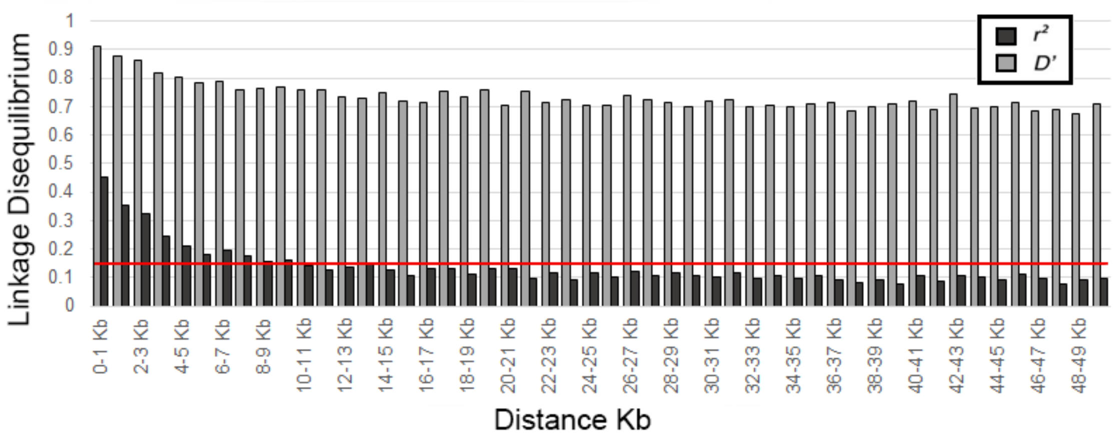

Figure S1. Genome-wide average linkage disequilibrium (LD) decay plot estimated across all chromosomes of *Eucalyptus* for the studied population. The LD values correspond to the average of the correlation between alleles at two loci (r^2) and the normalized disequilibrium coefficient (D') for each 1 Kpb. The LD threshold of r^2=0.14 is indicated with a red line.

Table S1. Summary of phenotypic information for quantitative traits related to wood quality and tree growth measured in a six-year-old breeding population of *E. globulus*.

Trait	Minimum	Maximum	Mean	Standard Deviation
Tree Height (m)	4.4	16.6	9.3	2.1
Diameter at breast height (cm)	3.3	25.3	11.6	3.1
Stem straightness (0 – 6)	0	6	-	-
Branch quality (0 – 6)	0	6	-	-
Wood density (mm)	10	23	16	2.0

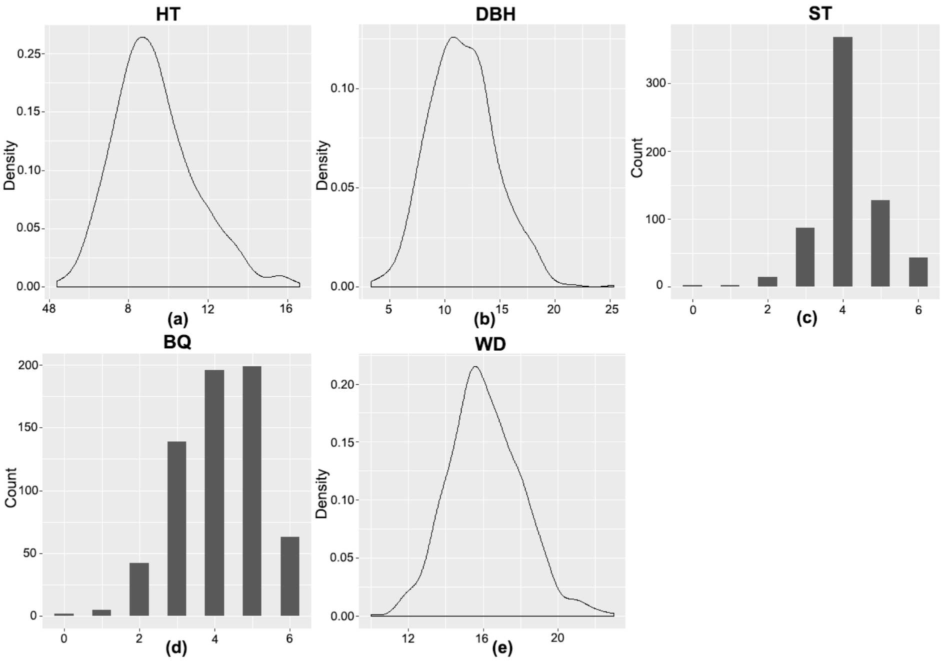

Figure S2. Distributions and histograms of the studied traits: (a) tree height (HT) in m, (b) diameter at breast height (DBH) in cm, (c) stem straightness (ST), (d) branch quality (BQ) and (e) wood density (WD) in mm.
